# Machine Learning Techniques for Chemical Identification Using Cyclic Square Wave Voltammetry

**DOI:** 10.3390/s19102392

**Published:** 2019-05-25

**Authors:** Scott N. Dean, Lisa C. Shriver-Lake, David A. Stenger, Jeffrey S. Erickson, Joel P. Golden, Scott A. Trammell

**Affiliations:** 1 National Research Council Postdoctoral Fellow, Washington, DC 20375, USA; scott.dean.ctr@nrl.navy.mil; 2 U.S. Naval Research Laboratory, Center for Bio/Molecular Science & Engineering (Code 6900), 4555 Overlook Avenue SW, Washington, DC 20375, USA; lisa.shriverlake@nrl.navy.mil (L.C.S.-L.); david.stenger@nrl.navy.mil (D.A.S.); jeffrey.erickson@nrl.navy.mil (J.S.E.); joel.golden@nrl.navy.mil (J.P.G.)

**Keywords:** electrochemical detection, cyclic square wave voltammetry, machine learning techniques

## Abstract

Electroanalytical techniques are useful for detection and identification because the instrumentation is simple and can support a wide variety of assays. One example is cyclic square wave voltammetry (CSWV), a practical detection technique for different classes of compounds including explosives, herbicides/pesticides, industrial compounds, and heavy metals. A key barrier to the widespread application of CSWV for chemical identification is the necessity of a high performance, generalizable classification algorithm. Here, machine and deep learning models were developed for classifying samples based on voltammograms alone. The highest performing models were Long Short-Term Memory (LSTM) and Fully Convolutional Networks (FCNs), depending on the dataset against which performance was assessed. When compared to other algorithms, previously used for classification of CSWV and other similar data, our LSTM and FCN-based neural networks achieve higher sensitivity and specificity with the area under the curve values from receiver operating characteristic (ROC) analyses greater than 0.99 for several datasets. Class activation maps were paired with CSWV scans to assist in understanding the decision-making process of the networks, and their ability to utilize this information was examined. The best-performing models were then successfully applied to new or holdout experimental data. An automated method for processing CSWV data, training machine learning models, and evaluating their prediction performance is described, and the tools generated provide support for the identification of compounds using CSWV from samples in the field.

## 1. Introduction

There is demand for automated, accurate, and fast identification of chemical threats and hazardous materials such as heavy metals, explosives, and herbicides/pesticides in the field. For this purpose, electrochemical detection provides a number of advantages including low-detection limits, amenability to miniaturization, and use of small analyte volumes [1]. One example is square-wave voltammetry (SWV), which is a powerful electrochemical technique practical for analytical and detection applications. Performing SWV experiments in a cyclic fashion can be viewed as an improved technique: cyclic SWV (CSWV), the very promising results of which have demonstrated improved peak resolution and increased sensitivity over traditional SWV [2].

A wide array of compounds can be identified using CSWV [3], where the performance for chemical identification within samples has been reported to rival high performance liquid chromatography [4] and other traditional chromatography techniques. The square-wave cathodic, anodic, and adsorptive stripping voltammetry are seen as modes that facilitate increasing pre-analysis concentration enrichment of the analytes and their determination down to the picomolar scale. CSWV has emerged as a viable low-cost alternative to many other expensive and time-consuming techniques for the identification of inorganic and biologically relevant compounds.

The typical potential waveform from CSWV consists of cathodic and anodic signals, which can provide a wealth of electrochemical information. In a manner similar to spectroscopy techniques, patterns found in CSWV data can be thought of as a fingerprint of the studied experimental system, which is both unique and consistent. Critical for utilization of CSWV fingerprints are classifier algorithms. Machine learning has been demonstrated to be valuable in CSWV, as in many other areas, for compound identification. Various machine learning techniques have been evaluated for their performance in the classification of CSWV datasets, many of which have been based on dimensionality-reduction techniques, including linear discriminant analysis (LDA) and principal component analysis (PCA). Specifically, LDA has been used with sensors to evaluate the polyphenolic content in olive oil [5], Liu et al. demonstrated the use of a PCA-support vector machine (PCA-SVM) for classification of green and black teas [6], and Ceto et al. utilized a PCA-artificial neural network ensemble for classifying a set of explosives [7]. More recently, Erickson et al. reported the use of a simple discrimination algorithm for the identification of nitrogen-containing explosives [8].

In this study, we sought to examine a range of algorithms that include more recently developed techniques. Surveyed were representative dimensionality-reduction techniques, such as LDA and PCA-SVM, as well as 1 Nearest Neighbor and Dynamic Time Warping (1NN-DTW) [9]. Also examined were more recently developed deep learning techniques, including Fully Convolutional Networks (FCNs) [10], which have been demonstrated to be powerful classifiers for time series data. Since CSWV data can be characterized as time series data, we hypothesized that these methods may be of practical use in this domain. Along with FCNs, we investigated simple Long Short-Term Memory (LSTM) networks, long-used as classifiers for a broad range of time series data [11], as well as a series of FCNs augmented by LSTM, which have been demonstrated to perform more robustly on many of the datasets within the University of California, Riverside (UCR) Time Series Repository [12]. In addition to a high level of performance, the convolutional layers within FCNs can yield information in the form of Class Activation Maps (CAMs), which provide insight into the decision-making process of the network, and has been shown to have potential utility in various diagnostic applications [13,14].

Here, we present the use of machine learning for easily automated, accurate, and fast identification of hazardous chemicals with a portable system for potential application in the field. Beyond this, we highlight some other favorable attributes of algorithms assessed in the study.

## 2. Materials and Methods 

### 2.1. Chemicals and Seawater Samples

Chemicals were purchased from Sigma-Aldrich and included CuSO_4_, PbCl_2_, HgCl_2_ and CdCl_2_, paraquat (PQ) and diquat (DQ), methyl parathion (MeP), Bisphenol-A (BPA) and nonyl phenol (NP). Seawater samples were obtained from the U.S. Naval Research Laboratory (Key West, FL, USA), the Marine Biological Laboratory (Vineyard Sound, MA) (unfiltered and filtered), bayside and oceanside (Ocean City, MD, USA; Honolulu, HI, USA) and oceanside (Duck, NC, USA). Field measurements were performed at bayside and oceanside (Ocean City, MD, USA; Honolulu, HI, USA) and oceanside (Duck, NC, USA).

### 2.2. Electrochemical Measurements

The electrochemical measures were made with a custom built hand-held potentiostat [8] using screen printed electrodes (SPEs) with a carbon working electrode (dimensions of 4 mm × 5 mm), a carbon counter electrode, and a Ag/AgCl reference electrode in a compact voltammetry cell purchased from Pine Research. The CSW voltammograms for seawater data sets were recorded at a frequency set to 17.5 Hz and an amplitude of 25 mV with a current range at 2 mA between 1.0 V and −1.0 V vs. Ag/AgCl with 2 min accumulation steps at 1.0 V and −1.0 V. All peak potentials (Ep) are reported as V vs. Ag/AgCl. Sample measurements were operated with a single button preprogramed with the CSW voltammogram parameters with back-to-back replicates.

### 2.3. Library and Sample Preparation

Library development from the electrochemistry assays for the explosive library set have been previously reported [8]. Results for the seawater assays were generated by spiking 10 mL of sample seawater with between 0 to 10 µL of stock solution of the chemical of interest such that we obtained sample concentrations between 50 and 2000 ppb. Two scans were recorded for each sample concentration and a new SPE electrode was used with each analyte. Samples included heavy metals, an organophosphate pesticide, herbicides, and industrial phenols.

### 2.4. Data Preprocessing 

From the developed library of samples, cathodic and anodic scans were concatenated (cathodic followed by anodic). Four datasets were created for training and testing: (i) 4-class chemicals in seawater (4-SW), (ii) 11-class chemicals in seawater (11-SW), (iii) 3-class explosives (3-EXP), and (iv) 11-class explosives (11-EXP). 4-SW was organized based on four categories: heavy metals (HM), phenols/industrial compounds (Ind), pesticides and herbicides (HandP), and seawater with no chemicals added (SW); an 11-SW dataset labeled each chemical separately: DQ, MeP, PQ, Cd, Cu, Hg, Pb, HM (heavy metal mixture of Cd, Hg and Pb), BPA, NP, SW. Similarly, the 3-EXP dataset containing nitroaromatics, nitramines, and nitrate esters was organized into three categories: a class (A) containing TNT, TNB, Tetryl, 2,6-DNT, 2,4-DNT, and 1,3-DNB, a second class (B) with 4-am, 2-am, 3-NT, and RDX, and a third class (C) containing buffer alone. The 11-EXP dataset listed each explosive compound separately. The training-test set splits were made such that each class retained approximately the same train-test split ratio of 70:30, 70:30, 70:30, and 50:50 for 4-SW, 11-SW, 3-EXP, and 11-EXP, respectively, using randomized, stratified, and shuffled training-test splits via the StratifiedShuffleSplit function from the Sci-Kit Learn library [15]. No additional preprocessing was performed on the datasets as they are near a mean of 0 and standard deviation of 1. The sample number of each class were as follows (class – number). For 4-SW: HM – 187, Ind – 38, HandP – 72, SW – 80. For 11-SW: DQ – 14, MeP – 50, PQ – 8, Cd – 36, Cu – 23, Hg – 22, Pb – 38, HMM – 68, BPA – 4, NP – 34, SW – 80. For 3-EXP: A – 36, B – 24, C – 6. For 11-EXP: six for each of the 11 classes.

### 2.5. Model Training

Seven models were tested on each seawater and explosives dataset. PCA-SVMs and LDAs methods were trained using the Sci-Kit Learn library [15], each with three components. The 1NN-DTW algorithm was implemented as described by Xi et al. [9] The FCN classifier was used and implemented by Wang et al. [10] and, for each FCN-containing algorithm, the FCN block was kept constant throughout the study, where the three 1-dimensional convolution layers had filter sizes of 128, 256, and 128, respectively, followed by global average pooling. The LSTM method with dimension shuffle was implemented as described by Karim et al. [12] and isolated from the rest of the architecture, without dropout. The LSTM-FCN and Attention (A)LSTM-FCN algorithms proposed by Karim et al. [12] were utilized as described. Two thousand training epochs were used to allow for convergence. The dropout rate of 80% was used after the LSTM/ALSTM layers to avoid overfitting. Class imbalance was handled via a class weighing scheme described by King and Zeng [16]. All neural network models were trained using the Keras [17] library with the TensorFlow [18] backend, via the Adam optimizer with an initial learning rate of 0.001 and a final learning rate of 0.0001. The learning rate was reduced by a factor of 2^1/3^ for every 100 epochs where no improvement was observed in validation accuracy, until the final learning rate (0.0001) was reached. All convolution kernels were initialized using the initialization proposed by He et al. [19]. All models were trained on an Ubuntu workstation with an Nvidia Geforce GTX1070 GPU.

### 2.6. Model Analysis and Refinement

F1-score with macro averaging, Receiver Operating Characteristic (ROC) curves, and ROC area under the curve (AUC) values were calculated using the classification report, ROC_curve, and AUC functions, respectively, from the Sci-Kit Learn library [15] metric module. F1-scores were calculated using a default threshold of 0.5. Following generation of the ROC curves, the AUC for each class was calculated, as well as the micro- and macro-averages. Repeated training-test splits (number of repeats: 25) were used for cross-validation [20]. Following the experiments, all data was organized and visualized in the statistical programming language R, making use of the tidyverse R package [21].

The optimal number of LSTM cells was determined by testing over a range from 128 to 4, where the cell number was halved successively, in which each model was trained for 500 epochs on each dataset at each cell number. The highest ROCAUC micro averages were used to determine the best cell number, for each dataset, and each LSTM model (LSTM, LSTM-FCN, and ALSTM-FCN). Batch size was optimized in the same manner as LSTM cell number, where the batch size was halved successively from 128 to 4. The optimal batch size for each dataset and model was chosen via the highest ROCAUC micro average. The optimization results for both batch size and LSTM cell number are represented as boxplots (from 5 repeated training-test splits). The parameters used after refinement are shown in Table S1. Summary boxplots are shown in Figure S1 (LSTM), Figure S2 (FCN), Figure S3 (LSTM-FCN), and Figure S4 (ALSTM-FCN). Values stated in Tables and Figures refer to the scores obtained by models after refinement.

Class Activation Maps (CAMs) were obtained and plotted as previous described [12], where values from the final 1D convolutional layer were visualized as heatmaps plotted over the original input.

## 3. Results

### 3.1. Cyclic Square Wave Voltammograms, Model Schematics and Data Processing

**Figure 1:**
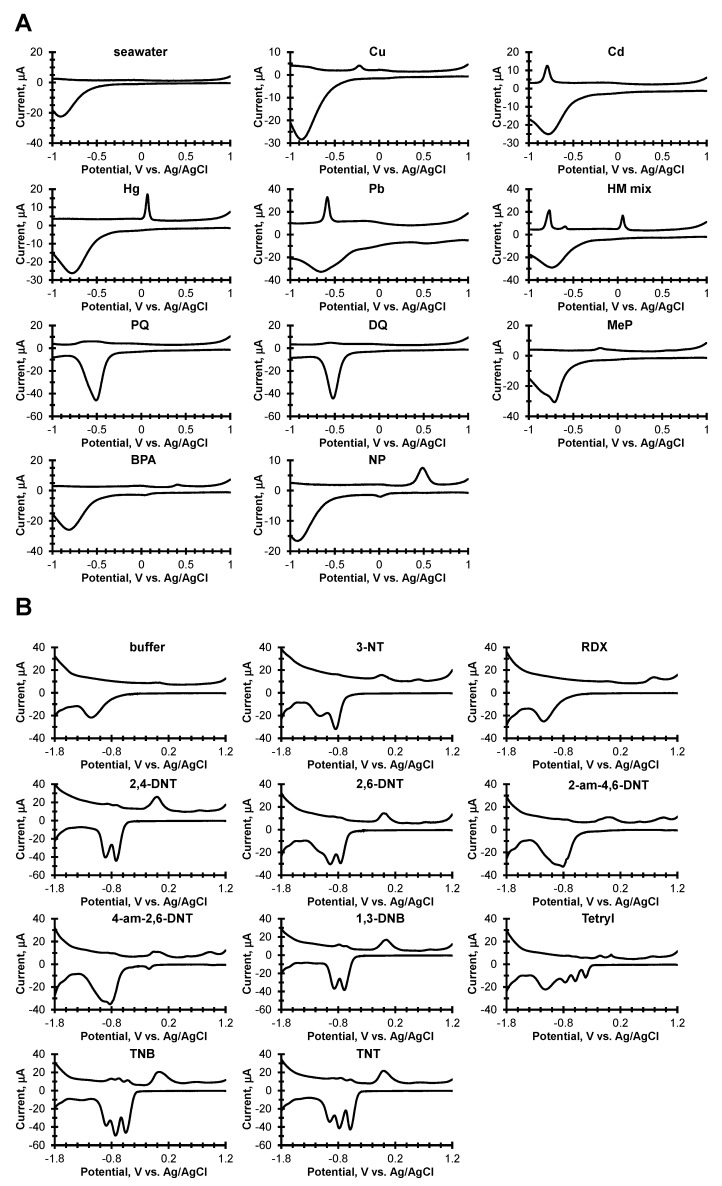
Electrochemical signatures for each compound. (**A**) Chemicals in seawater. (**B**) Explosives in buffer from reference [8].

Using the prototype instrument described by Erickson et al. [8], two libraries of explosives and seawater samples were compiled from CSW voltammograms from which four datasets were created for training and testing of the classifiers: 4-class chemicals in seawater (4-SW), 11-class chemicals in seawater (11-SW), 3-class explosives (3-EXP), and 11-class explosives (11-EXP). Dataset creation is described extensively in the Experimental section. Representative CSWV scans for each class are shown in Figure 1.

The CSWVs in Figure 1A show distinctive electrochemical signatures for each class of compounds. For example, CSWVs of seawater examples typically show an oxygen reduction in the cathodic scan with a peak position near −0.9 V vs. Ag/AgCl. For the various heavy metal cations, the cathodic peaks are concomitant with oxygen reduction, and the anodic stripping peaks have peak potentials at 0.06 V (Hg), −0.20 V (Cu), −0.58 V (Pb) and −0.78 V (Cd), vs. Ag/AgCl. The reduction of the herbicides, PQ and DQ show cathodic peaks at −0.51 V (PQ), −0.52 V (DQ) vs. Ag/AgCl, with small anodic board peaks centered at −0.55 V (PQ), and −0.56 V (DQ) vs. Ag/AgCl. A clear cathodic peak at −0.71 V vs. Ag/AgCl for MeP appears on the shoulder of the oxygen reduction. For BPA and NP, there are cathodic peaks at 0.036 V (BPA) and 0.016 (NP) vs. Ag/AgCl with two anodic peaks at 0.044 V and 0.41 V (BPA) and two anodic peaks 0.041 V and 0.48 V (NP) vs. Ag/AgCl.

**Figure 2:**
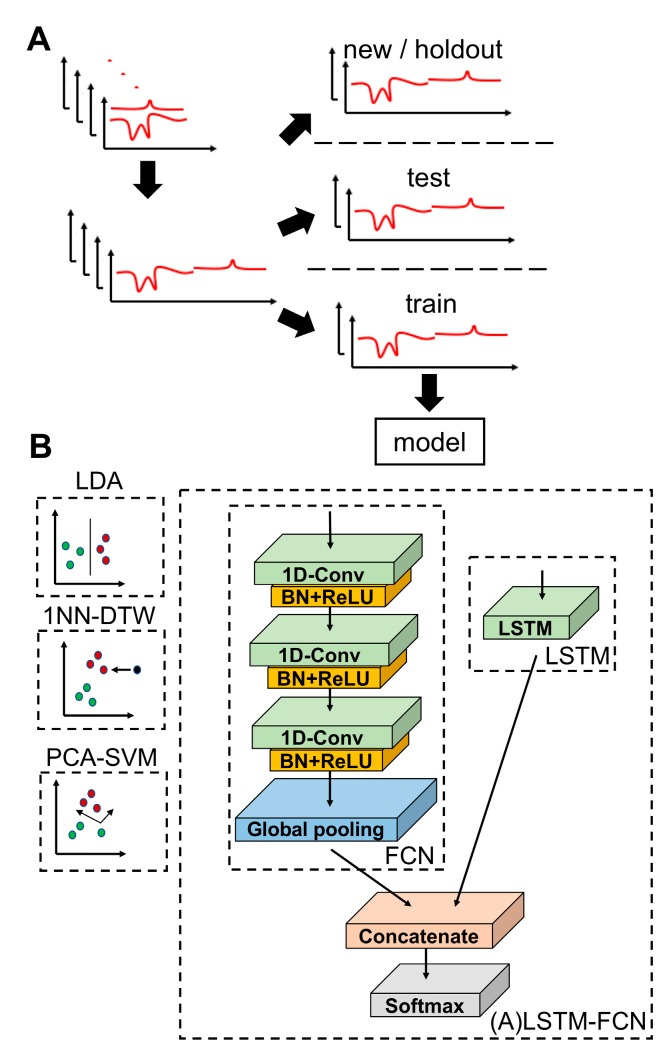
Data preprocessing and model schematics. (**A**) Schematic of data collection, preparation, and training. (**B**) Schematic representation of each algorithm is shown.

For machine learning analysis, a schematic representation of the data processing prior to training is displayed in Figure 2A. Beyond concatenation of cathodic and anodic scans for each sample, the experimental data was not modified. The training-test set splits were made such that each class retained approximately the same train: test split of 70:30, 70:30, 70:30, and 50:50 for 4-SW, 11-SW, 3-EXP, and 11-EXP, respectively.

Fivefold cross-validation was employed to obtain the optimal parameter values of each neural network classifier (LSTM, FCN, LSTM-FCN, and ALSTM-FCN) tested in the study, prior to performance comparison. In particular, two important parameters were optimized: LSTM cell number and batch size. For both, the ranges between 4 and 128 were run and the settings with the best cross-validation accuracy were selected. The final optimized LSTM cell number and batch size values are listed in Table S1 (more detailed depictions of ROCAUC results under each condition are supplied in Figures S1–S4). Of note was the dramatic decrease in classification performance under specific conditions for certain datasets, reinforcing the necessity for parameter optimization prior to classifier analysis when using the neural networks described.

As defined by the 1NN term of the 1NN-DTW algorithm, k was set to 1. The size of the warping window was set to 10. Unlike parameters above, where optimization was assessed over a large range, the warping window was limited by the large computational expense of running 1NN-DTW when the warping window parameter was set to larger values, forcing us to limit its assignment to values ≤ 10. The dimensionality of the PCA-SVM and LDA was set to 3, following parameter optimization over a range from 1 to 5, as values above 3 did not improve performance.

### 3.2. Comparison of the Classifiers

To improve upon the classification algorithm implemented in Erickson et al. [8] and CSWV classification algorithms described elsewhere, we tested seven different classifiers: PCA-SVM, LDA, 1NN-DTW, LSTM, FCN, LSTM-FCN, and ALSTM-FCN. A schematic representation of each model is displayed in Figure 2B. Each model was examined using the most performant parameterization described in the previous section. As an initial assessment of model performance, the macro harmonic means of precision and recall: the F1-score, were calculated from 25 repeated training-test splits, from which median and IQR were taken. Wilcoxon signed rank test was used to compare the median rank of each of the models. LDA and LSTM were found to have the highest median F1-scores on 11-EXP with 0.775 and 0.763, respectively, while the three FCN-containing models were more performant on 3-EXP with FCN, LSTM-FCN, and ALSTM-FCN tied at 1.000. For 11-SW, the top models were the four LSTM-augmented models achieving >0.96 where ALSTM-FCN was the highest at 0.985. Finally, for 4-SW, the four LSTM-augmented models were scored at > 0.95, with the highest being LSTM and FCN at 0.962. See Table 1 for the complete listing of median and IQR F1-score values. Importantly, the median F1-scores for each of the LSTM/FCN models on the two seawater datasets were significantly higher than each of the other models (PCA-SVM, 1NN-DTW, and LDA) examined (p < 0.05). Meanwhile, by F1-score, each of the models, with the exception of PCA-SVM, performed similarly on 11-EXP. On 3-EXP, the four LSTM-augmented models outperformed the others (p < 0.05).

Due to the relatively poor performance of both the PCA-SVM and 1NN-DTW classifiers, which were the two poorest-performing on three of the four datasets when compared to the other five classifiers (p < 0.05), both were excluded from further study. The exclusion of the standard 1NN-DTW was notable given its general citation as a reference algorithm for time series classification [22]; its poor performance may be related to a relatively low-warping window size necessitated by its long compute time required to reach completion (see Methods).

**Figure 3:**
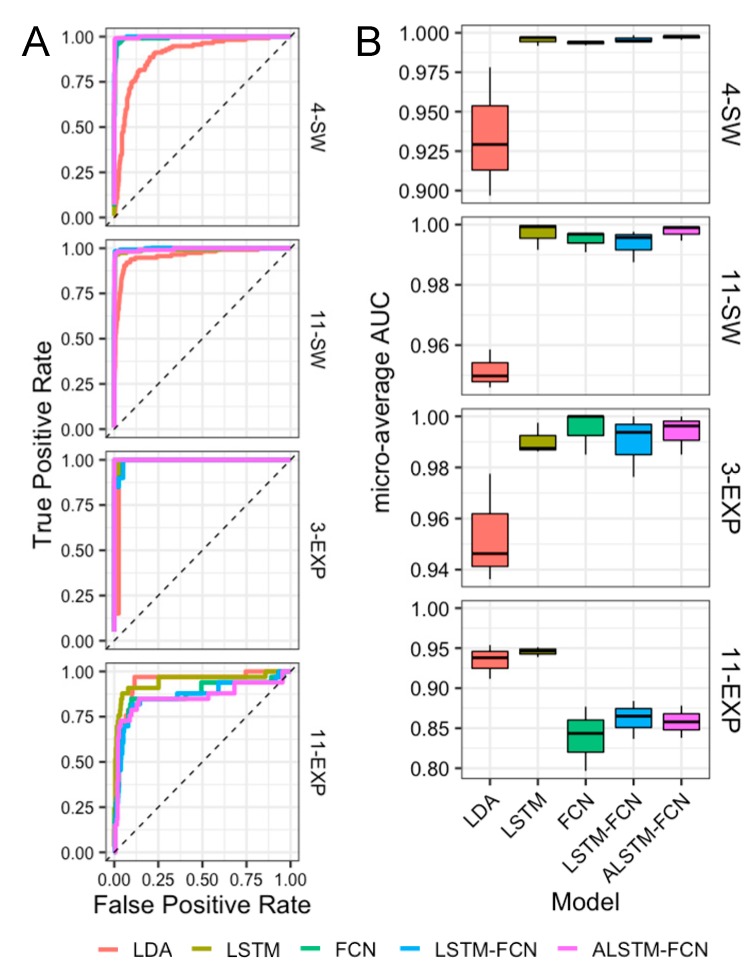
Receiver operating characteristic (ROC) curves and ROC area under the curve (ROCAUC) summary boxplots. (**A**) Representative micro-average ROC curves for each algorithm tested on each dataset in the study. (**B**) AUCs calculated from micro-average ROC curves were plotted from repeated (≥ 25) training-test splits for each algorithm.

Since the macro-average F1-scores with a fixed classification threshold are relatively incomplete indicators of classification performance particularly on datasets with class imbalances (here, 4-SW and 11-SW), we sought to further investigate classifier performance using a second evaluation metric: Receiver Operating Characteristic (ROC) curves. Representative micro-average ROC curves for each algorithm tested on the 4-SW, 11-SW, 3-EXP, and 11-EXP datasets are shown in Figure 3A. Both relatively high true positive (TPR) and 1-false positive rates (1-FPR) for all models were seen for each of the datasets with sufficient sample size per class, whereas noticeably lower TPR and 1-FPR is seen in the case of 11-EXP, which contains 3-samples per class in the training and test sets. For each of the datasets, 11-EXP excluded; the ROC curves of LSTM, FCN, LSTM-FCN, and ALSTM-FCN were largely overlapping, making interpretation difficult and necessitating use of the area under the curve (AUC) values. ROCAUCs were calculated from micro-average ROC curves making use of repeated (25 replicates) training-test splits for each algorithm on each of the four datasets. Boxplots of these results are shown in Figure 3B, tested on each dataset in the study. From this analysis, the highest median of the micro-average ROCAUCs achieved for each dataset were as follows: 1.000 on 3-EXP using FCN, 0.999 on 11-SW using ALSTM-FCN, 0.995 on 4-SW using ALSTM-FCN, and 0.946 on 11-EXP using LSTM. For each of the datasets except 11-EXP, the four LSTM models yielded significantly higher micro-average ROCAUC values than LDA (p < 0.05). In this study, 11-EXP was found to be the most challenging for the models utilized, where LSTM and LDA were the best performing models with median ROCAUC values of 0.946 and 0.938, respectively. Interesting was the relatively high level of performance by LDA, which may result from the smaller sample size in 11-EXP which likely has a larger negative impact on deep learning models. ROCAUC summary data (median and IQR) are provided in Table 2.

### 3.3. Class Activation Mapping 

The FCN-containing models used in the study (FCN, LSTM-FCN, and ALSTM-FCN) allow for visualization of the class activation map (CAM), while ALSTM models are capable of providing concept vectors, both of which can be exploited for showing regions that are most influential towards the classification decision. Given this interesting property of FCNs and the CAMs they can provide, the usefulness of CAMs for study of the CSWV electrochemical fingerprint of individual samples was examined.

**Figure 4:**
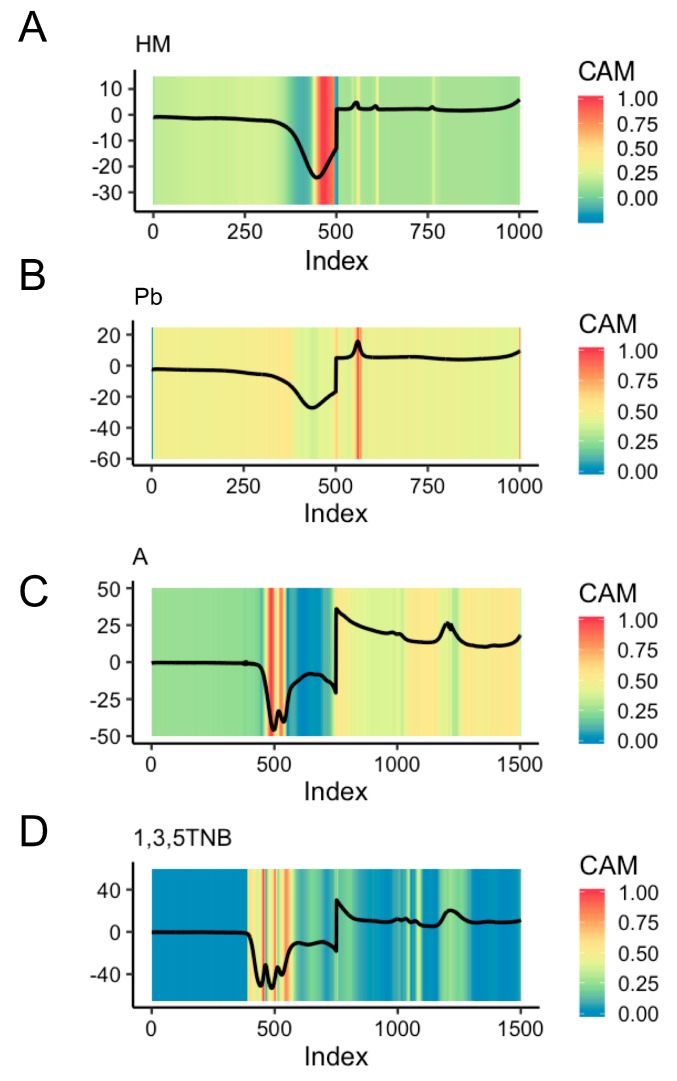
Information that fully convolutional network (FCN)-containing models provide. (**A**) Class activation map (CAM) for 4-SW. Representative HM sample is shown. (**B**) CAM for 11-SW. Representative Pb sample is shown. (**C**) CAM for 3-EXP. Representative A sample is shown. (**D**) CAM for 11-EXP. The representative 1,3,5-TNB sample is shown.

Representative CAMs for individual classes within the four datasets are shown in Figure 4, where each of the CAMs are resulting from the best-performing FCN-containing models for each dataset: LSTM-FCN for 11-EXP, FCN for 3-EXP, and ALSTM-FCN for both 11-SW and 4-SW. In the HM (heavy metals) example from 4-SW, the regions found to be indicative for classification were found at index ~450–500 within the cathodic scan and three anodic stripping peaks (Figure 4A). This individual HM sample corresponds to an HMM (heavy metal mixture) sample in the 11-SW dataset. The region highlighted within the cathodic scan represents the reduction of oxygen overlapped with the reduction of metal ions. The three anodic stripping peaks picked out by the FCN as deciding factors for HM classification, at ~560 (−0.768 V vs. Ag/AgCl), ~610 (−0.584 V vs. Ag/AgCl), and ~770 (0.072 V vs. Ag/AgCl) are peaks corresponding to Cd, Pb, and Hg, respectively.

In the representative Pb CAM for 11-SW (Figure 4B), the main discernable region of interest for the ALSTM-FCN used was the anodic stripping peaks at the index of ~560 (−0.768 V vs. Ag/AgCl), previously noted in the HMM sample. Beyond this, the only other feature highlighted of note was at the very end of the anodic scan at index ~1000 (0.992 V vs. Ag/AgCl). It is unclear whether this is an experimental artifact or a feature relevant to Pb. However, since the CAM is recognizing it as an activator for the classification of this sample as Pb, it may be worthy of more investigation. 

The representative CAM for Class A of the 3-EXP dataset from the FCN shown in Figure 4C indicates most attention was paid toward the two well-resolved cathodic peaks in the region of index ~450–550, representing the number of nitro groups in 1,3-DNB (which in this individual sample corresponds to in the 11-EXP dataset). Since Class A is composed of scans containing nitro-aromatics each with clear cathodic peaks as their predominant attribute, the model may uniformly categorize these as “A”. Also of note was the lack of activation in the indices below 450 (−0.6 V vs. Ag/AgCl) and the increased interest in the region surrounding the concatenation point at ~750 (−1.8 V vs. Ag/AgCl).

Similarly, for 11-EXP, the representative CAM for a 1,3,5-TNB sample (Figure 4D) also largely highlights the peaks in the cathodic scan indicative of nitro groups at ~500 (−0.8 V vs. Ag/AgCl). Regions of interest to the classifier in the anodic scan, particularly the anodic peaks at ~1010 (−0.768 V vs. Ag/AgCl) and ~1230 (0.112 V vs. Ag/AgCl) may be muted due to its presence in several other classes within 11-EXP (Figure S10), lessening its importance as a discriminative attribute. Other examples of attributes identified by generated CAM visualization are in the supplemental material: 4-SW (Figure S5), 11-SW (Figure S6), 3-EXP (Figure S7), and 11-EXP (Figure S8). Of note is the appearance of CAMs from classes containing little information such as the buffer or seawater, which has high activation values for nearly the entire dataset; consistent with CAMs produced for decisions made on other datasets [13].

Interestingly, certain attributes identified in the scans as important for classification by the CAMs were not readily identifiable as being important in expert analysis (see Figure 4B and other examples in Figures S5–S8). In these instances, it remains possible that the ALSTM-FCN model is highlighting regions of importance that can be attributed to experimental artifacts or noise that was consistent throughout the samples within a particular class; however, the model may be detecting regions that are generally ignored by visual analysis that may have an electrochemical association specific to the sample class.

**Figure 5:**
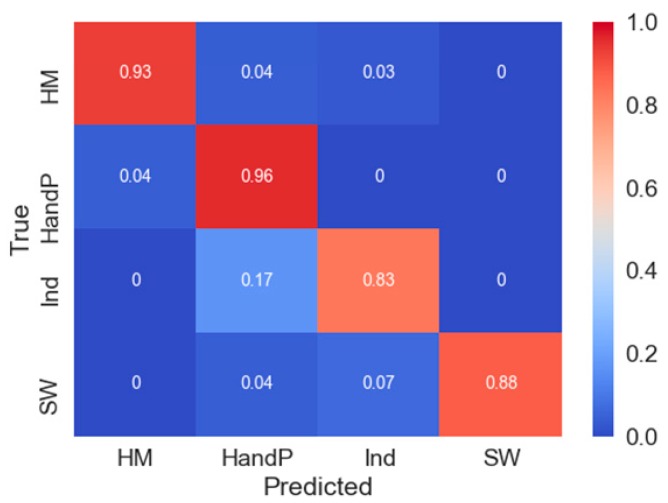
Application of CAM information with truncated dataset. Confusion matrix demonstrating retained classification ability of the truncated dataset, following truncation of the 4-SW dataset from 1002 in length to 400 (where the first 502 and last 100 data points were removed).

Finally, as a simple assessment of CAMs utility, we truncated the 4-SW dataset. Using the CAM from each of the classes of heavy metals (HM), herbicides and pesticides (HandP), industrial phenols (Ind), and seawater (SW) (Figure S5), the decision-making process of the highest-performing model—in the case of 4-SW, ALSTM-FCN—is concentrated around the middle of the concatenated dataset. Using the CAM as a guide, the new smaller 4-SW dataset was created by trimming 4-SW down from 1002 to 400 (in terms of index size), removing the first 502 and last 100 data points resulting in a dataset largely only retaining the anodic stripping peaks found in the chemicals in seawater samples. Following training on this new truncated dataset, the confusion matrix when evaluated on testing data demonstrates largely retained classification ability of the LSTM-FCN (Figure 5), when compared to the best performing LSTM-FCN model on the entire dataset (Figure S9A). Overall, this simple example suggests that CAMs may be useful as a form of filtering of CSWV data for content that is deemed necessary for classification.

### 3.4. Evaluation of Best Classifier on New Datasets 

**Figure 6:**
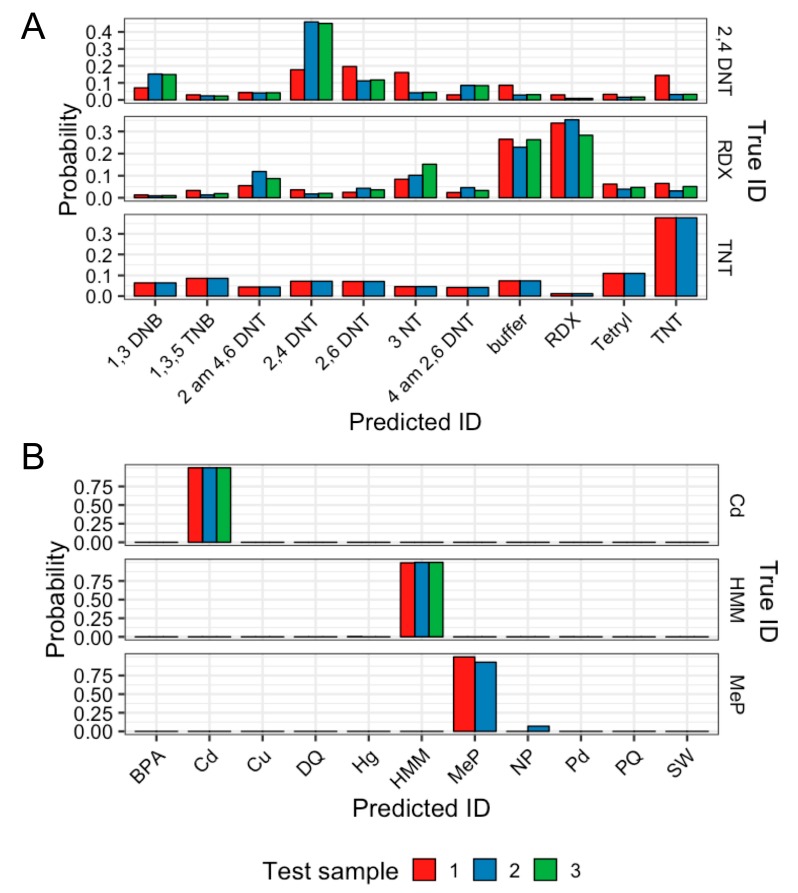
Evaluation of best model on a new experimental dataset of explosives samples or holdout seawater data. (**A**) Eight scans from three random classes were held out from training and tested for a final evaluation. Evaluated using ALSTM-FCN, the best performing model for classification of seawater with 11 classes. Eight out of eight samples were correctly classified (**B**) Scans from each compound (10 µg) wiped off benchtop. Evaluated using LSTM, the best performing model for classification of explosives with 11 classes. Seven out of eight samples were classified correctly, with Sample #1 of 2,4 DNT classified as 2,6 DNT.

The best-performing models for 11-SW and 11-EXP were evaluated on two small (n = 8) datasets as a final assessment of real-world classification performance. The data was either held out from 11-SW, where eight scans were selected at random from three classes, or was newly obtained experimental data, which was the case for validation of the 11-EXP dataset (Figure 6). Using median ROCAUC values, ALSTM-FCN obtained the highest for 11-SW (0.999) and LSTM was found to be the highest for 11-EXP (0.946).

The more performant ALSTM-FCN model successfully correctly classified each sample, with no second-choice probability higher than 0.1 (Figure 6A). This result was consistent with the perfect classification observed in some of the training-test splits (Figure S9B). In contrast, the performance of the LSTM on 11-EXP yielded 7 out of 8 correct assignments, with one 2,4-DNT sample incorrectly assigned as 2,6-DNT (Figure 6B). Notable was the higher uncertainty of the predictions the model provides on the 11-EXP data, possibly related to the smaller overall sample size for the 11-EXP dataset, relative to 11-SW, and associated lower ROCAUC values for the evaluated models on 11-EXP. 

Overall, the results were most impressive on both tests and holdout or new experimental data for the chemicals in seawater data, where we also saw promising results when the classes of 11-SW were further split based on concentration, into 25 classes total (Figure S9C). However, concentration-level classification was not thoroughly investigated in this study, and results suggested higher sample numbers might be necessary for further study.

## 4. Discussion

This study made use of our previously reported potentiostat described by Erickson et al. [8], with the aim to enhance automated identification. An essential component and natural next step toward this goal is the development of generalizable CSWV data classifiers. Here, we sought to improve upon chemical identification algorithms proposed in previous studies [7,8] and develop library-independent models that can be used to correctly identify chemical samples in the field using CSWV data, with the hope to enable a more automated classification process.

In order to assess the performance of established and cutting-edge machine learning and deep learning algorithms; including baseline algorithms used in previous reports for classification of CSWV samples, we exploited the time series structure of CSWV data where current is measured in a series of regular intervals while adjusting potential. With this approach, we made use of previously developed and optimized algorithms for time series data classification. In addition to a PCA-SVM benchmark for classification of CSWV samples, we examined some common algorithms used for classification of time series including LDA which has been utilized in both cheminformatics [23] and time series classification more generally, with 1NN-DTW, a benchmark algorithm for time series classification [9,22], as well as numerous deep learning techniques.

Deep learning algorithms—which are comprised of layers of neural networks—have been repeatedly demonstrated to provide state-of-the-art performance for classification tasks [24]. Here, we employed both recursive neural networks (LSTMs), convolutional neural networks (FCNs), and a combination of the two, as described by Karim et al. [12], where FCNs are augmented by LSTMs. Although, PCA-SVMs have been previously reported to provide promising performance for the classification of some CSWV data [6], in our study it delivered poor performance in validation accuracy, F1-scores, and ROCAUC values following test data evaluation on each of the four datasets, relative to the other algorithms examined. Generally used as a gold standard time series classifier, the 1NN-DTW algorithm provided a strong baseline in comparison. However, similar to the PCA-SVM results, it performed relatively poorly in our study. LDA demonstrated surprisingly strong classification performance on the 11-EXP, in particular. The small size of the 11-EXP dataset (n = 6 per class) is likely the cause of the higher performance of LDA relative to the deep learning models, known to require more training data for optimal learning, relative to many other machine learning techniques.

FCNs and FCN-containing classifiers have previously been shown to have a high-level baseline performance on a wide array of time series data [10]. Since the performance of FCNs and neural network classifiers more broadly suffer from overfitting with small datasets such as the datasets used here, such as the relatively small 11-EXP dataset, there is reason to believe their performance can be improved with a larger number of samples per class. Assessment of promising classifiers from this study on larger datasets will be the subject of future investigation for CSWV data classifier development.

Comparing classification performance via ROCAUC has certain advantages over F1-scores, including the characteristics of scale and classification-threshold-invariance. When compared by ROCAUC, the results for LSTM and FCN models obtained very high levels of performance by ROCAUC scores, nearing perfect, on both test and holdout or new experimental data for the chemicals in seawater data. As the seawater sample library was larger in total and on a sample-per-class basis, we cannot be sure whether the higher scores seen when assessed on the seawater data relative to the explosives is due to differences in data or dataset size. Since both Wang et al. and Karim et al. found FCNs and FCNs augmented with LSTMs to have generalizable performance on the UCR Time Series Repository data [10,12], we believe the classifier performance immediately improve when provided with more experimental samples.

In addition to promising classification performance, properties of FCNs and ALSTMs can be exploited for extracting more information from CSWV data and allow practitioners to understand the decision-making process of the model. For FCNs, we extracted the activation maps for regions that are most influential towards the classification decision. By looking up the corresponding expert annotations for those regions, we were able to see which nameable visual phenomena contributed to the network’s final classification [25]. Previous application of CAMs included the highlighting of mammogram images for cancerous lesion identification [14] and identification of chemicals in spectroscopic magnetic resonance imaging data [13]. Unlike in image classification, and unique to time series data, information provided by visualizing CAMs can be used for the truncating datasets, either during or post data collection. The immediate usefulness of this may include the ability to run shorter scans, requiring less data and faster data analysis. The size of the data required to train the model has implications for both the size of the network and model. Both a smaller model footprint on the hard drive and a smaller and faster network required for classification may be valuable for practical usage in the field where classification speed is essential, and especially where device miniaturization means hard drive space is at a premium.

Beyond practical utility, visualization of CAMs can be contrasted with expert annotation of CSWV data for basic science considerations. For example, the peaks corresponding to −0.768, −0.584, and ~770 (V versus Ag/AgCl), in the heavy metal mixture samples were quickly identifiable by expert as corresponding to Cd, Pb, and Hg, respectively. However, the certain extraneous highlighted regions at 0.992 V vs. Ag/AgCl apparent in the CAMs of the Pb sample anodic scans were not readily identifiable. Whether these regions are most important for the classification decision are artifacts from data collection or signals corresponding to real phenomena that generally go unnoticed, requires further investigation. For ALSTMs, the attention mechanism allowed for visualization of the decision process of the LSTM cell, which may provide additional information to the practitioner beyond that of the CAM.

Although training most deep learning models such as those in this study can be very computationally intensive and require powerful, expensive hardware, querying the generated models in the field would be significantly less computationally intensive. For use in the field, we developed a Python application to query the model for individual samples. Using a trained model, this application performed inference CSWV scans on an affordable computer, such as those with a small form-factor (e.g., Raspberry Pi), which can be suitably paired with our inexpensive, portable potentiostat. In addition, the best performing models have been deployed to an in-house website application to allow for them to be easily applied in ongoing experiments.

## 5. Conclusions

This study demonstrates the successful use of various machine learning and deep learning models to classify chemicals from CSWV data. In particular, deep learning algorithms previously determined to be strong in the classification of general time series data were successfully applied to CSWV data and found to have a high level of performance. Moreover, the information gleaned from FCN-containing models in the form of CAMs was demonstrated to provide additional useful material, including allowing for dataset truncation, while retaining classification ability. Also notable was the identification of the strong baseline classifier for CSWV data in the relatively simple LDA, especially in our data-limited datasets. Despite these impressive results—there is still room to improve performance with the deep learning methods applied CSWV-based classification, of great potential for sample identification in biological and chemical systems, will likely see increasingly impressive performance and wider employment in various fields, with access to larger amounts of data, a wider array of sample types, and increased focus on actionable information obtainable in CAMs, each of which will be the subject of future studies.

## Figures and Tables

**Table 1:** F1-score (median of macro-averages and interquartile-range (IQR)) following cross-validation.

Model	11-EXP		3-EXP		11-SW		4-SW	
	Median	IQR	Median	IQR	Median	IQR	Median	IQR
PCA-SVM	0.335	0.069	0.587	0.093	0.190	0.051	0.233	0.025
1NN-DTW	0.712	0.226	0.923	0.043	0.680	0.058	0.735	0.015
LDA	0.775	0.090	0.923	0.167	0.842	0.010	0.803	0.105
LSTM	0.763	0.074	0.960	0.040	0.965	0.012	0.962	0.027
FCN	0.679	0.016	1.000	0.040	0.961	0.010	0.962	0.018
LSTM-FCN	0.687	0.060	1.000	0.040	0.975	0.012	0.953	0.030
ALSTM-FCN	0.697	0.061	1.000	0.040	0.985	0.006	0.960	0.033

**Table 2:** Receiver operating characteristic area under the curve (ROCAUC) summary.

Model	11-EXP		3-EXP		11-SW		4-SW	
	Median	IQR	Median	IQR	Median	IQR	Median	IQR
LDA	0.938	0.021	0.946	0.021	0.950	0.006	0.929	0.041
LSTM	0.946	0.006	0.988	0.006	0.999	0.004	0.997	0.003
FCN	0.843	0.040	1.000	0.008	0.997	0.003	0.994	0.001
LSTM-FCN	0.865	0.024	0.994	0.012	0.996	0.005	0.995	0.002
ALSTM-FCN	0.858	0.020	0.996	0.008	0.999	0.002	0.998	0.001

## Supplementary Materials

The following are available online at https://www.mdpi.com/1424-8220/19/10/2392/s1, Table S1: LSTM cell number and batch size parameters for neural networks in the study, Figure S1: Batch size and LSTM cell number optimization for LSTM, Figure S2: Batch size for FCN, Figure S3: Batch size and LSTM cell number optimization for LSTM-FCN, Figure S4: Batch size and LSTM cell number optimization for ALSTM-FCN, Figure S5: Representative CAMs of each class in SW-4, Figure S6: Representative CAMs of each class in SW-11, Figure S7: Representative CAMs of each class in EXP-3, Figure S8: Representative CAMs of each class in EXP-11, Figure S9.: Confusion matrix for (A) 4-SW, (B) 11-SW, and (C) 11 SW datasets.
