# Supplementary material for: Machine Learning Techniques for Chemical Identification Using Cyclic Square Wave Voltammetry
Source: Sensors (Basel). 2019 May 25;19(10):2392. doi: 10.3390/s19102392 (PMC6567068; doi:10.3390/s19102392)
Supplement: Supplementary file 1 [file sensors-19-02392-s001.pdf]

Machine learning techniques for chemical identification using cyclic square wave voltammetry

Scott N. Dean, Lisa C. Shriver-Lake, Dave A. Stenger, Jeffrey S. Erickson, Joel P. Golden, and Scott A. Trammell*

Supporting information

		LSTM	FCN	LSTM-FCN	ALSTM-FCN
11-SW	BS	128	4	4	8
	LCN	128	NA	4	8
4-SW	BS	128	4	8	16
	LCN	128	NA	128	128
11-EXP	BS	128	4	4	4
	LCN	128	NA	4	16
3-EXP	BS	128	4	16	4
	LCN	128	NA	16	16

Table S1. LSTM cell number and batch size parameters for neural networks in the study. Both the optimal LSTM cell number (LCN) and batch size (BS) were determined following repeated training-test split cross validation.

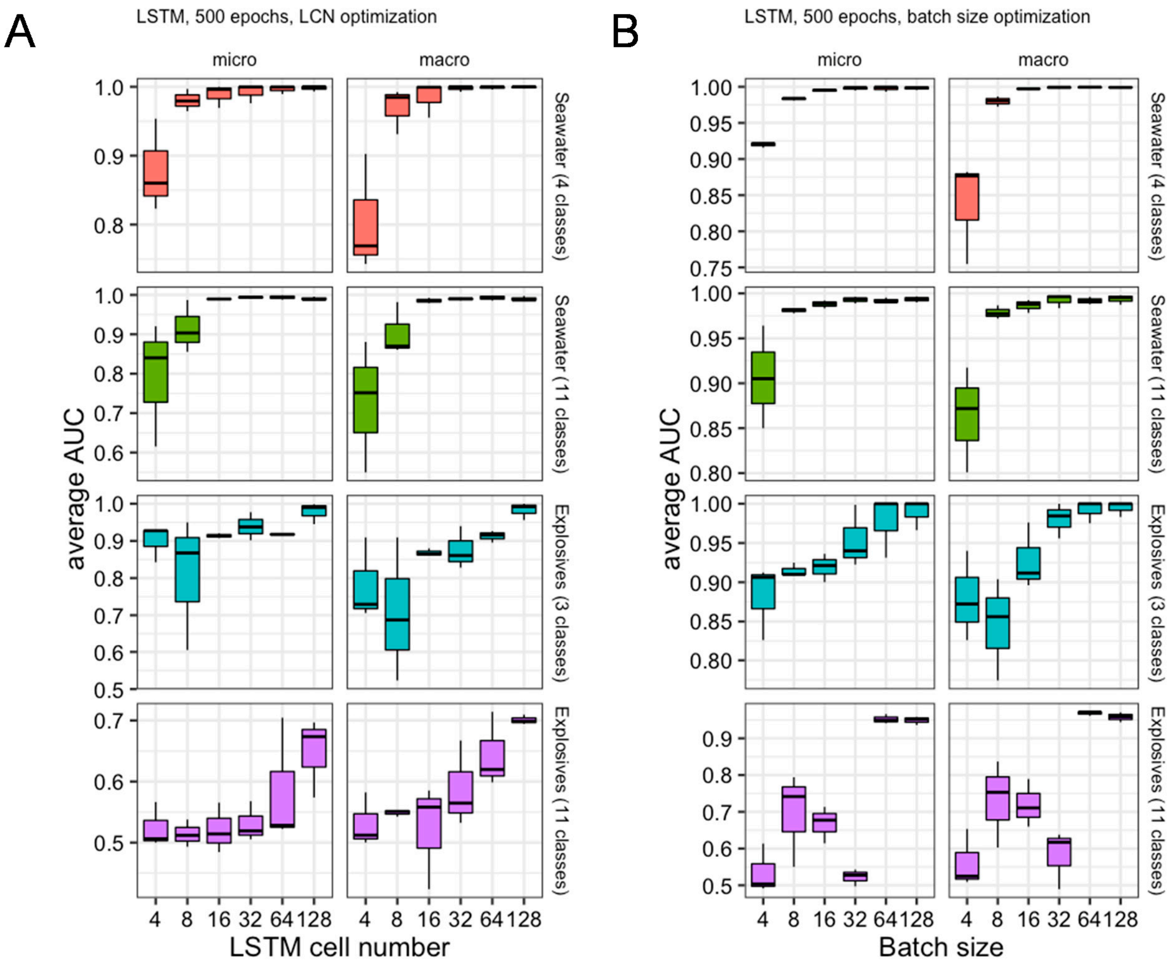

Figure S1. Batch size and LSTM cell number optimization for LSTM. (A) Differing batch sizes were tested using LSTM over 500 epochs. Micro- and macro-average ROCAUCs after repeated training-test splits were plotted. (B) Differing LSTM cell number were tested using LSTM over 500 epochs. Micro- and macro-average ROCAUCs after repeated training-test splits were plotted.

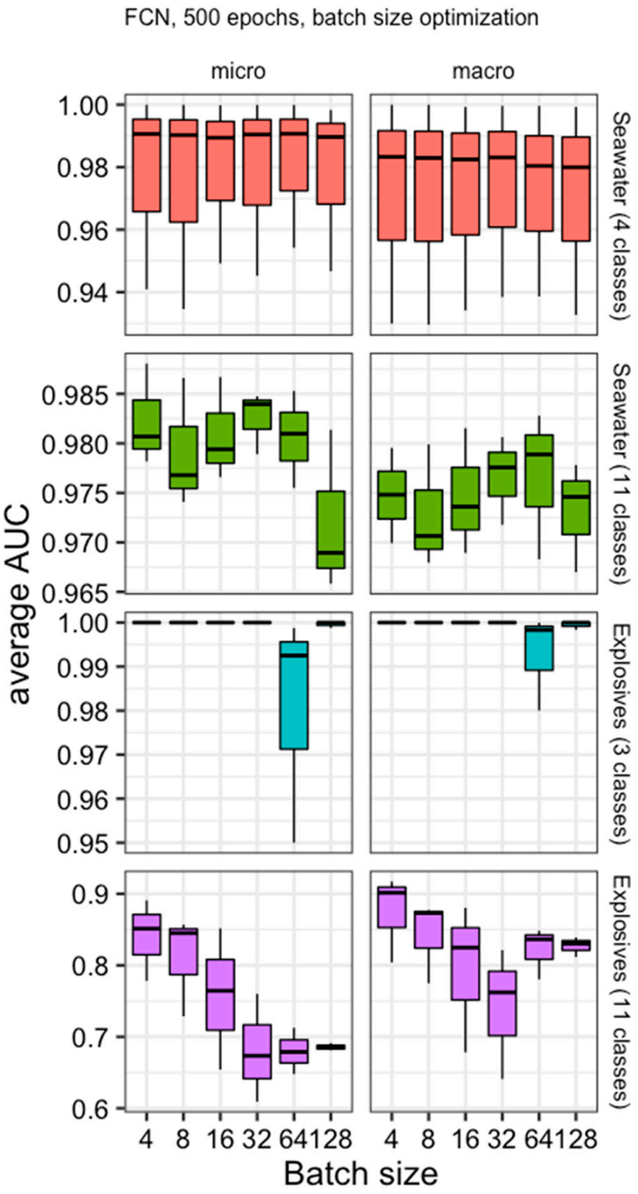

Figure S2. Batch size for FCN. Differing batch sizes were tested using FCN over 500 epochs. Micro- and macro-average ROCAUCs after repeated training-test splits were plotted.

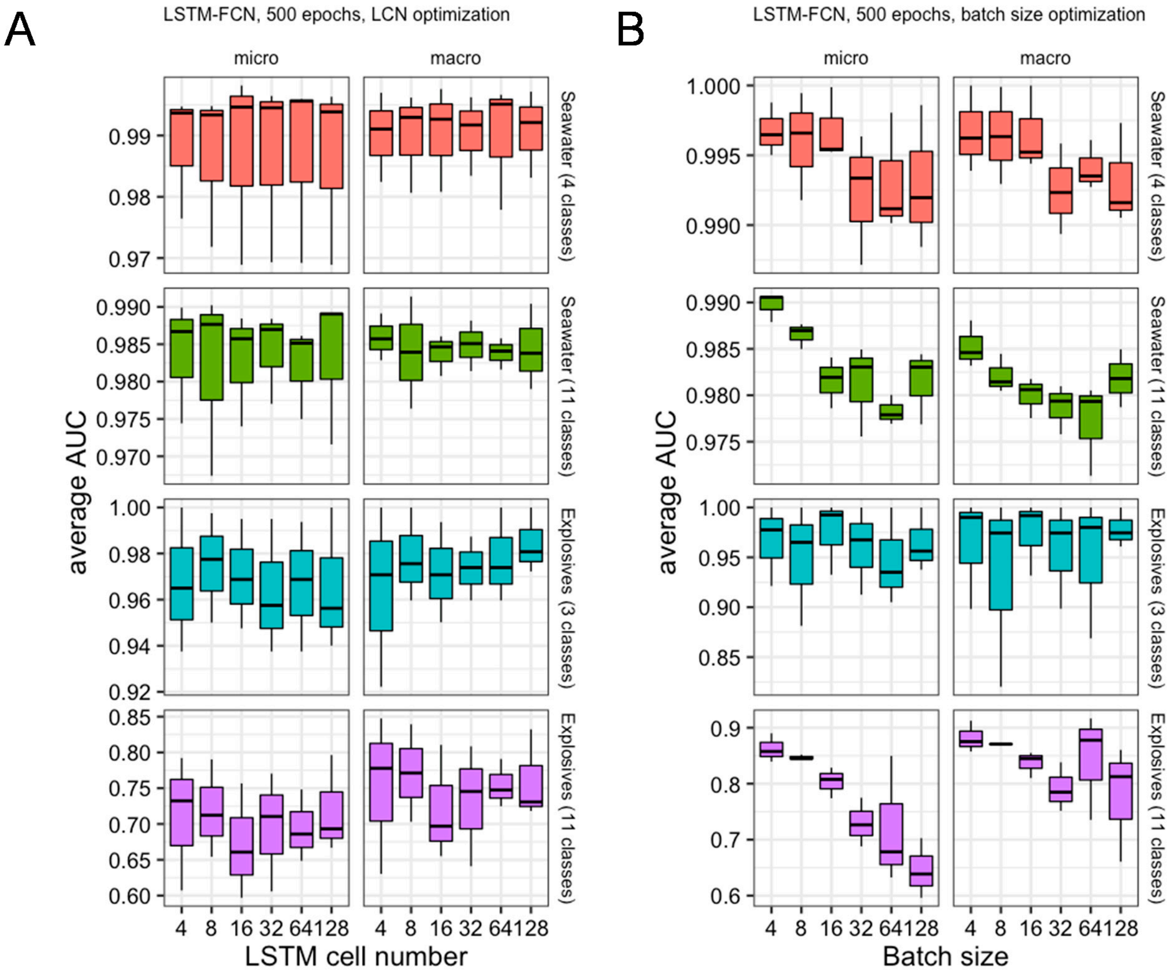

Figure S3. Batch size and LSTM cell number optimization for LSTM-FCN. (A) Differing batch sizes were tested using LSTM-FCN over 500 epochs. Micro- and macro-average ROCAUCs after repeated training-test splits were plotted. (B) Differing LSTM cell number were tested using LSTM-FCN over 500 epochs. Micro- and macro-average ROCAUCs after repeated training-test splits were plotted.

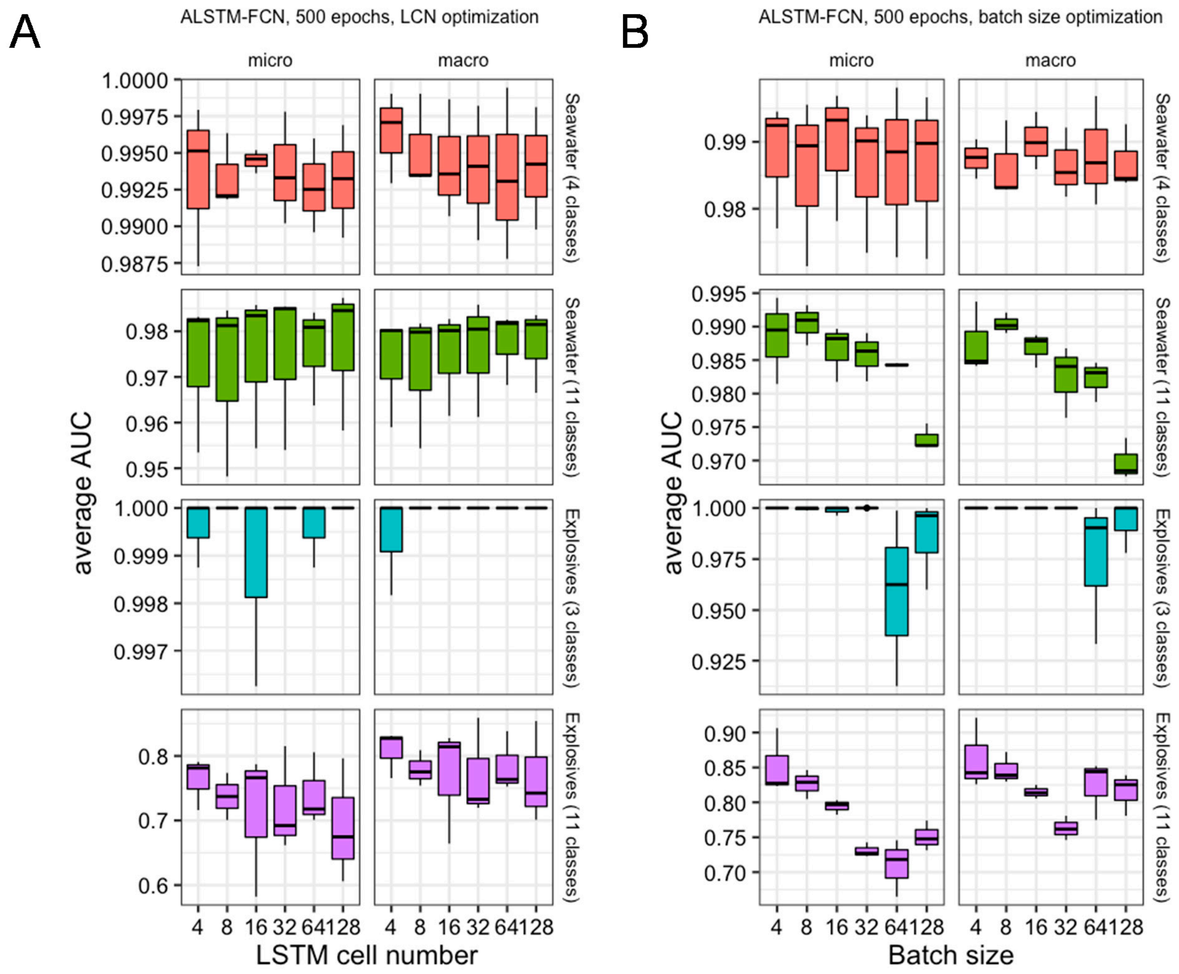

Figure S4. Batch size and LSTM cell number optimization for ALSTM-FCN. (A) Differing batch sizes were tested using ALSTM-FCN over 500 epochs. Micro- and macro-average ROCAUCs after repeated training-test splits were plotted. (B) Differing LSTM cell number were tested using ALSTM-FCN over 500 epochs. Micro- and macro-average ROCAUCs after repeated training-test splits were plotted.

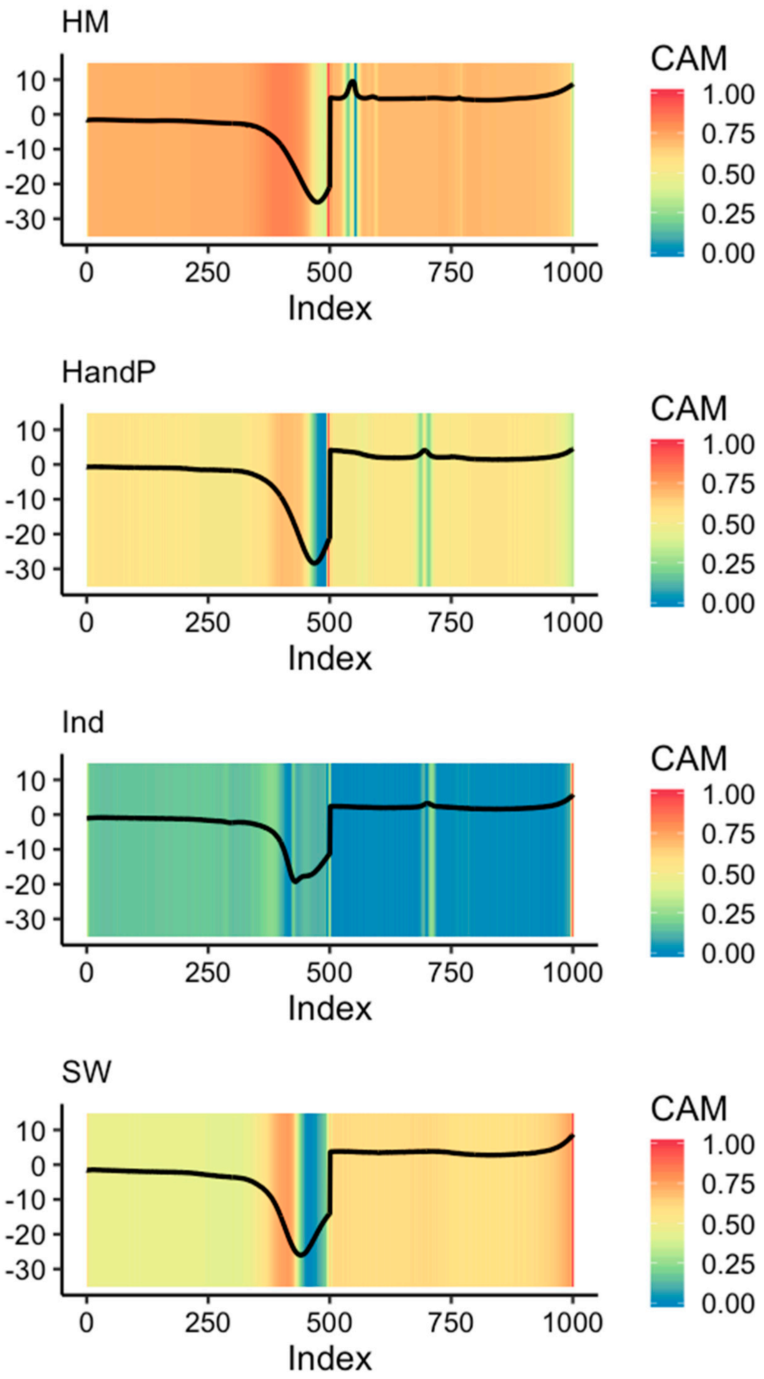

Figure S5. Representative CAMs of each class in SW-4. Label definitions: Heavy metals (HM), phenols / industrial compounds (Ind), pesticides and herbicides (HandP), and seawater with no chemicals added (SW).

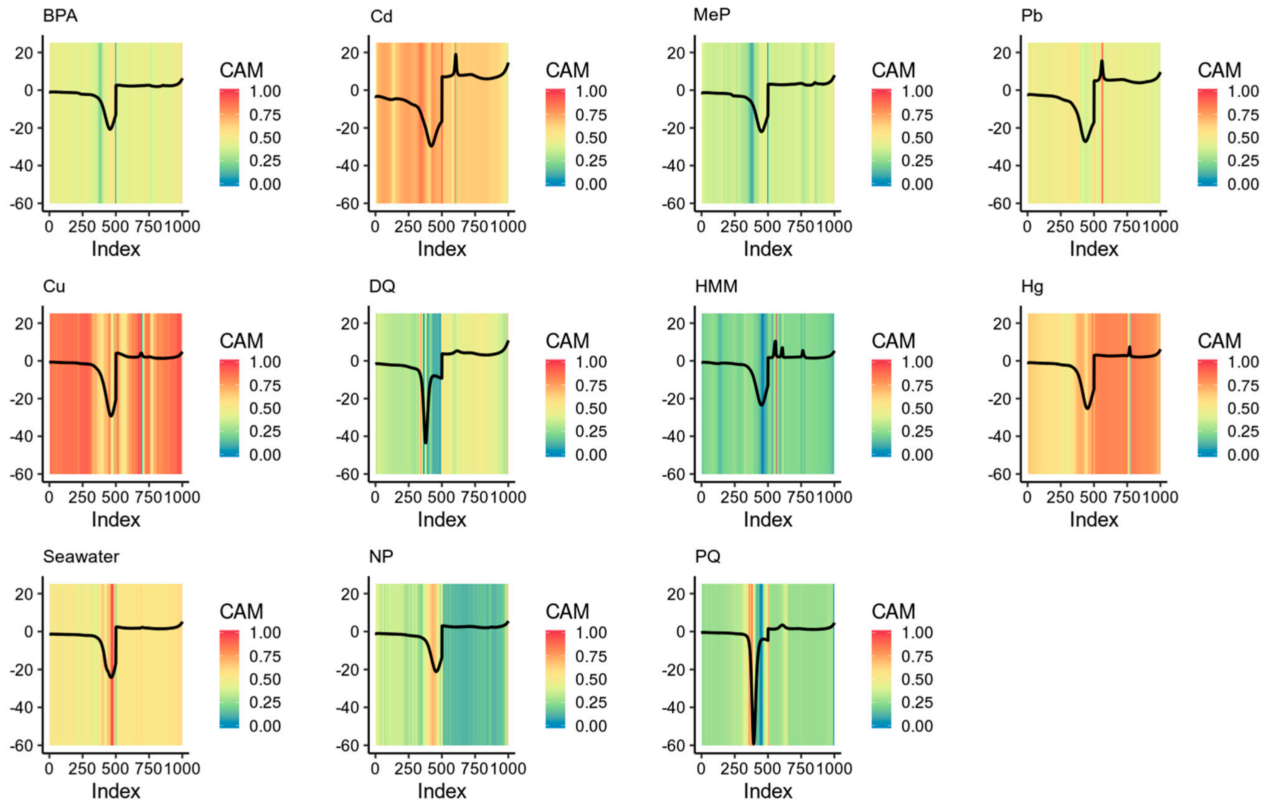

Figure S6. Representative CAMs of each class in SW-11. Label definitions: $CuSO_4$ (Cu), $PbCl_2$ (Pb), $HgCl_2$ (Hg) and $CdCl_2$ (Cd), paraquat (PQ) and diquat (DQ), methyl parathion (MeP), Bisphenol-A (BPA), nonyl phenol (NP), and HMM (heavy metal mixture of Cd, Hg and Pb).

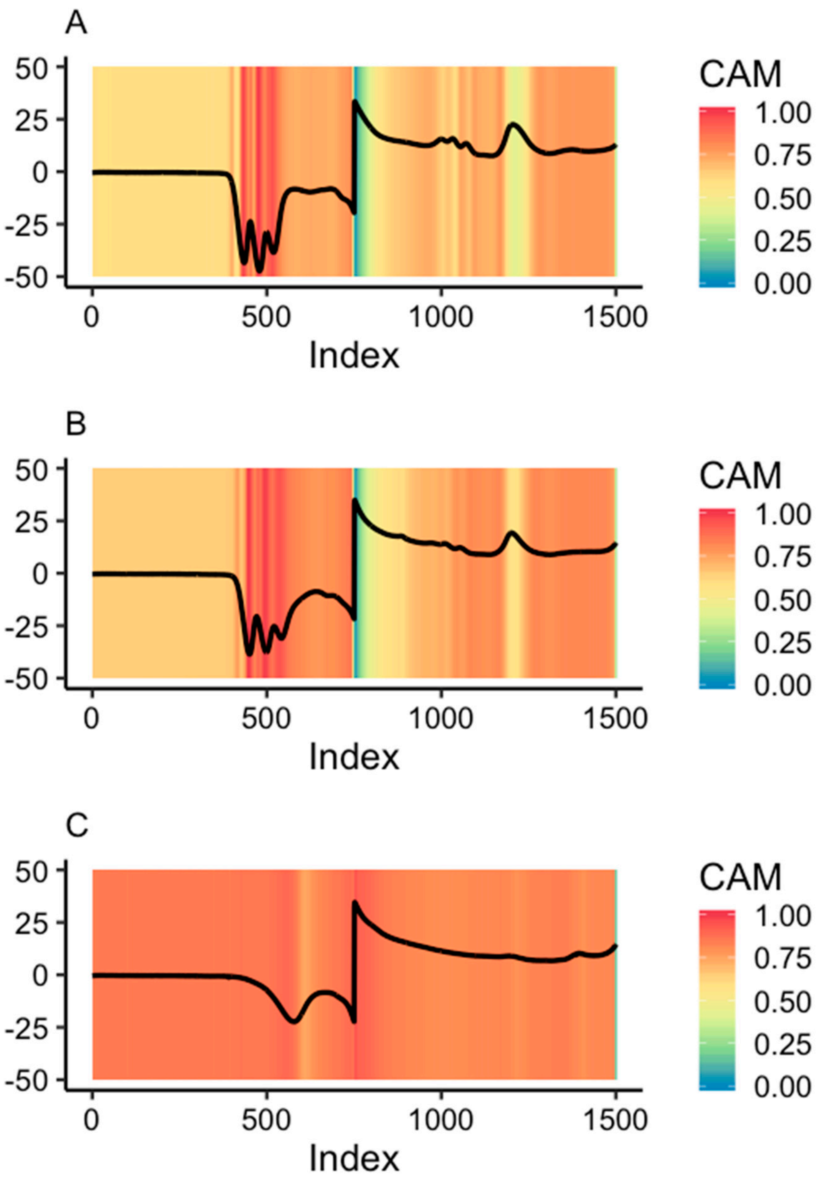

Figure S7. Representative CAMs of each class in EXP-3. Label definitions: class A containing TNT, TNB, Tetryl, 2,6-DNT, 2,4-DNT, and 1,3-DNB; class B containing 4-am, 2-am, 3-NT, and RDX; class C containing buffer alone.

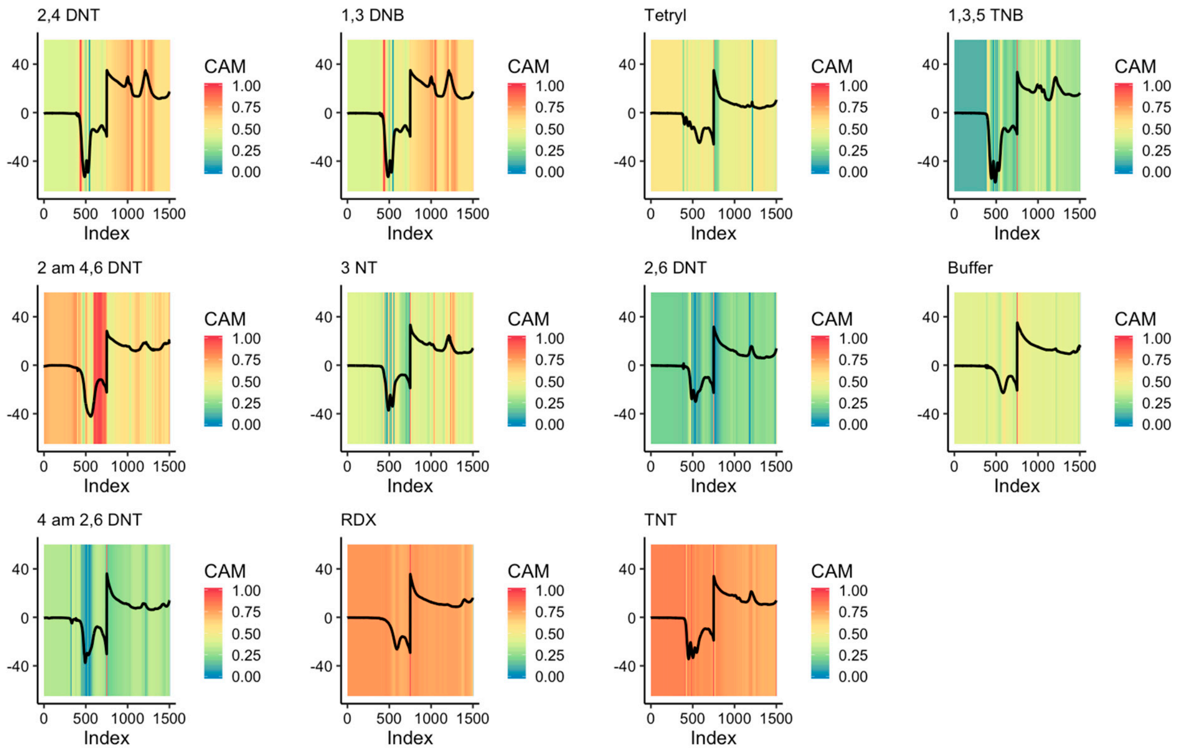

Figure S8. Representative CAMs of each class in EXP-11. Explosive library set previously reported.[8]

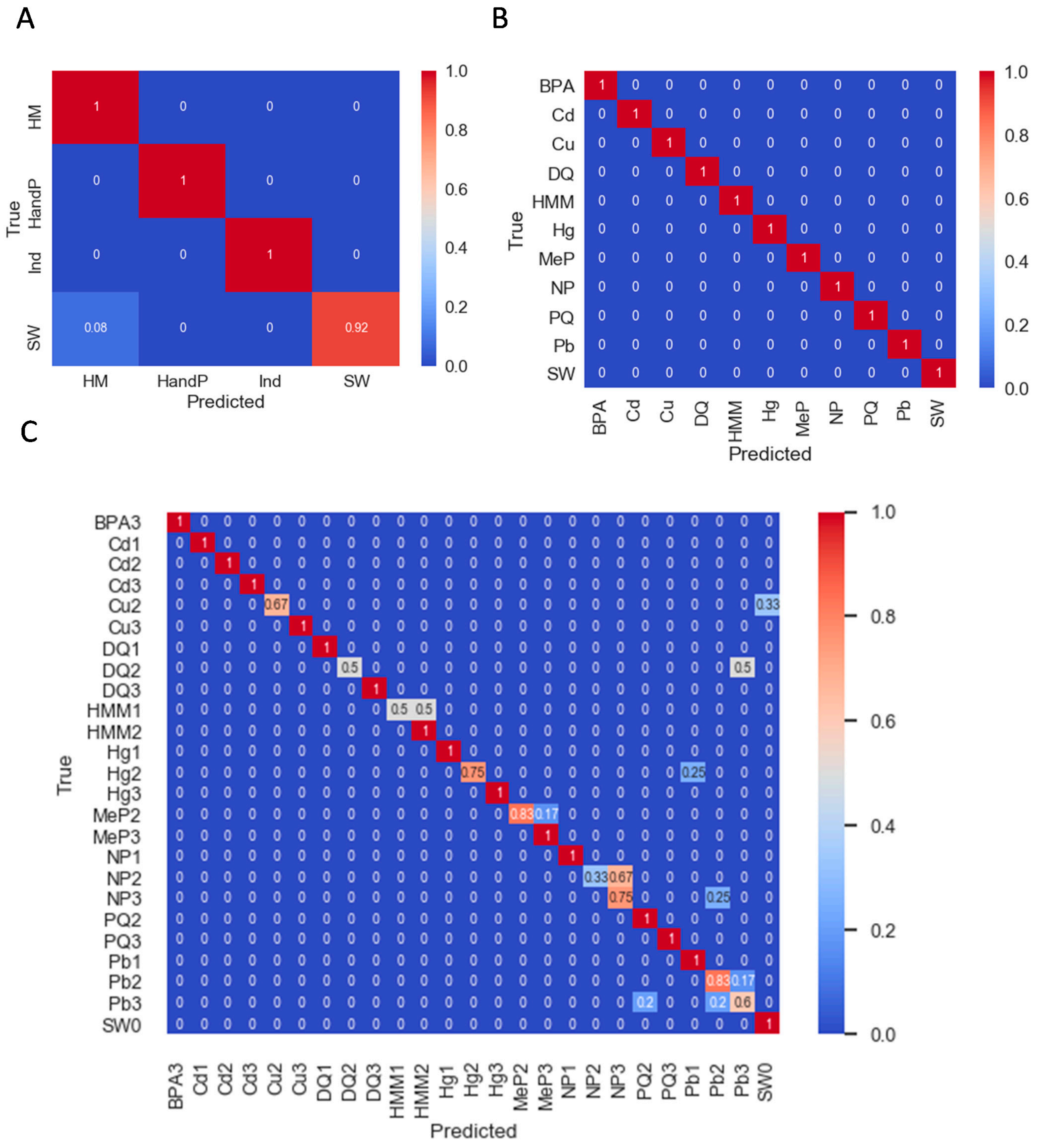

Figure S9. Confusion matrix for (A) 4-SW, (B) 11-SW, and (C) 11 SW dataset when separated by concentration range (25 classes total), using the best LSTM-FCN or ALSTM-FCN models. Label definitions: Figure S9A. Heavy metals (HM), phenols / industrial compounds (Ind), pesticides and herbicides (HandP), and seawater with no chemicals added (SW); Figure S9B. $CuSO_4$ (Cu), $PbCl_2$ (Pb), $HgCl_2$ (Hg) and $CdCl_2$ (Cd), paraquat (PQ) and diquat (DQ), methyl parathion (MeP), Bisphenol-A (BPA), nonyl phenol (NP), and HMM (heavy metal mixture of Cd, Hg and Pb). Figure S9C. Similar labels but with 1, 2 or 3 corresponding to concentration ranges: 1 < 100 ppb, 2 = 100 – 750 ppb and 3 > 750 ppb.
